# Droplet Microfluidic Technology for the Early and Label-Free Isolation of Highly-Glycolytic, Activated T-Cells

**DOI:** 10.3390/mi13091442

**Published:** 2022-09-01

**Authors:** Claudia Zielke, Adriana J. Gutierrez Ramirez, Kelsey Voss, Maya S. Ryan, Azam Gholizadeh, Jeffrey C. Rathmell, Paul Abbyad

**Affiliations:** 1 Department of Chemistry and Biochemistry, Santa Clara University, Santa Clara, CA 95053, USA; 2 Department of Pathology, Microbiology, and Immunology, Vanderbilt University Medical Center, Nashville, TN 37232, USA; 3 Vanderbilt Center for Immunobiology, Vanderbilt University Medical Center, Nashville, TN 37232, USA

**Keywords:** microfluidics, droplet microfluidics, cytometry, sorting, passive sorting, T-cells, metabolism, glycolysis

## Abstract

A label-free, fixation-free and passive sorting method is presented to isolate activated T-cells shortly after activation and prior to the display of activation surface markers. It uses a recently developed sorting platform dubbed “Sorting by Interfacial Tension” (SIFT) that sorts droplets based on pH. After polyclonal (anti-CD3/CD28 bead) activation and a brief incubation on chip, droplets containing activated T-cells display a lower pH than those containing naive cells due to increased glycolysis. Under specific surfactant conditions, a change in pH can lead to a concurrent increase in droplet interfacial tension. The isolation of activated T-cells on chip is hence achieved as flattened droplets are displaced as they encounter a micro-fabricated trench oriented diagonally with respect to the direction of flow. This technique leads to an enrichment of activated primary CD4+ T-cells to over 95% from an initial mixed population of naive cells and cells activated for as little as 15 min. Moreover, since the pH change is correlated to successful activation, the technique allows the isolation of T-cells with the earliest activation and highest glycolysis, an important feature for the testing of T-cell activation modulators and to determine regulators and predictors of differentiation outcomes.

## 1. Introduction

T-cells are a type of lymphocyte whose activation is considered a critical step in the adaptive immune response [1]. The activation of CD4+ T-cells leads to the proliferation and differentiation of T-helper (Th) subtypes that both control the immune response via cytokine secretion and directly attack infected or cancerous cells [2]. The full activation of T-cells requires the engagement of the T-cell receptor (TCR) and co-stimulation of CD28 by antigen presenting cells (APC), such as a dendritic cell [3]. Polyclonal stimulation via antibody-conjugated beads mimics this dendritic cellular interaction by presenting both CD3 and CD28 stimulatory antibodies colocalized on the bead surface [4]. The strength of TCR signaling during CD4+ T-cell activation regulates T-cell differentiation into T helper (Th) subsets such that a strong signal favors pro-inflammatory Th1 cell differentiation and a weaker signal promotes Th2 cells [5]. Additionally, weak TCR signal strength also promotes the differentiation of anti-inflammatory regulatory T cells (Treg) by limiting signaling through the mammalian target of rapamycin (mTOR), a key metabolic regulator of T-cell differentiation [6]. Cellular metabolism and glycolysis in particular, is now understood to be a key regulator of T-cell activation and differentiation which is also influenced by TCR signal strength [5,7,8].

Although metabolic reprogramming after T-cell activation is central to determining T-cell fate and function among Th subsets, the early events that dictate these metabolic changes within minutes of TCR stimulation have been difficult to measure in heterogeneous samples [9]. The interplay between metabolism on early activation and the differentiation of T-cells remains poorly understood, partly due to the lack of single-cell technology to capture cells based on their metabolic profiles [10]. The conventional methods of isolating activated T-cells rely on the use of antibodies that are specific to activation surface markers (such as CD25, CD69 and CD71) [11]. However, it can take many hours and as much as a full day after activation for specific activation markers to be displayed and detectable, greatly limiting fast detection and selection [12]. Alternatively, secreted cytokines such as IL-2 and IL-10, may be detectable within a couple of hours of activation, but require the fixation and manipulation of the golgi apparatus with monensin or brefeldin A for their detection [13,14]. This chemical inhibition of cytokine secretion is irreversible and renders cells unviable for downstream studies [15]. Encapsulating cells in microfluidic droplets enables the confinement of cytokines within a picoliter droplet [16,17,18,19]. These techniques provide the high throughput and sensitivity of fluorescent sorting. However, they require a prior knowledge of the target cytokine and specific fluorescent probes which can vary among different Th subsets, for detection and sorting [20].

In contrast to the display of activation surface markers or cytokines, changes in cellular metabolism upon TCR activation present a general indicator of activation [21] and occur within minutes after the activation of T-cells [7,22]. Naive T-cells attain most of their energy needs through oxidative phosphorylation [8]. However, upon activation, T-cells undergo metabolic reprogramming to support rapid replication and effector functions [21,23,24]. This anabolic and bioenergetic demand is covered by enhanced metabolism, in particular glycolysis [21,23,24]. Moreover, this metabolic switch is not simply a downstream product of activation but rather a key regulator of activation and T-cell differentiation [24,25]. The ramp up of glycolysis where glucose is converted to lactate, otherwise known as aerobic glycolysis or the “Warburg effect” [26], is a characteristic shared by many proliferating cells including cancer cells [27]. Increased glycolysis leads to the secretion of protons and lactate rendering the surroundings of the cells more acidic [28]. The increased glycolysis and its concurrent extra-cellular acidification upon activation presents a unique handle for the early isolation of activated T-cells. For these reasons, there is a large interest in the immunometabolism field for new methods that can isolate cells based on their active metabolic state as opposed to traditional activation markers. A recent method designed to profile heterogenous immune cell samples based on their metabolic profile and glycolytic metabolism is SCENITH flow cytometry [29], which uses metabolic inhibitor treatments followed by puromycin to quantify translation as a readout for the energy state of the cells. Although useful for profiling metabolic dependencies at the single-cell level, this methodology also requires the fixation of cells for the detection of intracellular puromycin and leaves samples unviable for downstream studies.

Microfluidics has been used extensively for single cell studies in immunology including to profile T-cell signaling dynamics, [30,31,32] isolate cells of interest [16,17,18,33] or study cell–cell interactions [34,35]. Droplet microfluidics is often used for these applications, as it is well adapted to the manipulation of suspended cells and confines secreted molecules at high concentrations [16,32,36,37,38]. Our lab recently developed a novel sorting technique dubbed “Sorting by Interfacial Tension” (SIFT) based on the observation that under specific chemical conditions, a decrease in pH leads to a concurrent increase in droplet interfacial tension [39]. This technique was previously used to sort enzymes based on activity, [40], photo-tagged droplets [41], empty and cell-occupied droplets, live and dead cells [39], and cancer cell subpopulations [42]. By leveraging changes in metabolism upon activation, the use of SIFT is demonstrated here for the early isolation of highly activated T-cells from naive cells. This method offers both label-free and passive sorting to separate activated T-cells in minutes rather than hours after activation. Passive sorting has been used in the literature to describe both techniques that select droplets without external fields (electric, acoustic, optical etc.) [43] and also those that do not use electronics for active droplet selection [44]. SIFT would fit both these definitions of passive sorting. Given the links between metabolism with activation and differentiation, SIFT offers a method to isolate activated T-cells based on their active metabolic state in real-time without chemical manipulations or fixation. This proof of concept is important for the immunometabolism field as isolating viable T cells within minutes of activation may be used to determine regulators and/or predictors of differentiation outcomes.

## 2. Materials and Methods

*Jurkat T-Cell Activation and Preparation for on-chip experiments:* Jurkat, Clone E6-1 TIB-152™ Human Acute T-cell leukemia cells were purchased from ATCC and grown at 37 °C in a 5% CO_2_ atmosphere in ATCC-formulated RPMI-1640 Medium supplemented with 10% fetal bovine serum (HyClone, GE Healthcare Life Sciences, Logan, UT, USA) and 2% *v*/*v* penicillin–streptomycin (10,000 units/mL–10,000 μg/mL) solution (Gibco, Life Technologies Corporation, Grand Island, NY, USA).

On the day of experiment, Jurkat T-cells were centrifuged, washed with 1X PBS and resuspended in PBS. To distinguish cell populations, naive control cells were labeled with Calcein AM (Thermo Fischer, Waltham, MA, USA), a viability fluorescent dye, for 30 min at 37 °C and 4% CO_2_ atmosphere. Subsequently, the cells were washed again and resuspended in media at a cell concentration of 1 × 10^6^ cells/mL.

Gibco™ Dynabeads™ Human T-Activator CD3/CD28 for T-Cell Expansion and Activation (Thermo Fisher Scientific, Waltham, MA, USA) were washed 3x with media and suspended at a bead concentration of 1 × 10^6^ beads/mL to ensure a 1:1 bead to cell ratio after mixing. To activate T-cells, beads and cells were mixed and incubated at 37 °C in an CO_2_ incubator for varying times. Alternatively, cells were also activated with soluble activation complexes (ImmunoCult, StemCell Technologies, Vancouver, BC, Canada) following manufacturer protocol. After activation, cell/bead suspensions were pipetted up and down to disturb the bead/cell aggregates and beads were separated from the cells using a magnet (Invitrogen DynaMag^TM^-2, Thermo Fisher Scientific, Waltham, MA, USA). Care was taken to ensure that the naive cells, which were not exposed to activation beads, experienced the same mechanical manipulations and washing steps as the activated cells.

Naive and activated cell populations were prepared separately, centrifuged, and resuspended in on-chip solutions. Naive and activated cell suspensions were prepared at a final cell concentration of about 5 × 10^5^ cells/mL which was determined using a Cellometer Auto T4 Bright Field Cell Counter (Nexcelcom Bioscience LLC, Lawrence, MA, USA) to ensure single cell occupation of droplets. On-chip solutions were a 1:1 mix of media and 1X PBS buffer. The media was prepared without fetal bovine serum (deproteinated media), but both solutions were supplemented with 1% *w*/*w* Pluronic F-68 (Affymetrix Inc., Maumee, OH, USA), 15% *v*/*v* Optiprep (Fresenius Kabi Norge AS for Axis-Shield PoCAS, Oslo, Norway) and 0.1 mg/mL pyranine (AAT Bioquest Inc., Sunnyvale, CA, USA). Solution pH and osmolality (determined with Vapro Vapor Pressure Osmometer 5520, Wescor, ELITech Biomedical Systems, Logan, UT, USA) of on-chip solutions were adjusted to physiological values (pH 7.4; 280–320 mOsmol) prior to experiment. Pluronic F-68 was used to promote droplet stability and cell viability, whereas Optiprep modulated solution density to limit cell sedimentation within the tubing and droplets. Pyranine served as a fluorescent ratiometric pH probe for analyzing droplet pH on chip. Before injection onto chip, activated and naive cell suspensions were combined at approximately a 1:1 ratio.

*CD4+ T-Cell Activation and Preparation for on-chip experiments:* Cryo-preserved, fresh CD4+ Helper T-Cells from a healthy donor were purchased from HemaCare Corporation, Northridge, Los Angeles, CA, USA. Cells were thawed, washed and resuspended in media as described above, and incubated for 2 h to allow the cells to recover. Subsequently, CD4+ T-cells were activated and prepared for on-chip experiments as described above.

*Cell Treatment with 2-Deoxy-D-glucose (2DG):* Jurkat cells were harvested and suspended in low glucose media supplemented with 100 mM 2DG (Sigma Aldrich, St. Louis, MO, USA) whereas a Calcein AM labeled control population was resuspended in the same media with 100 mM glucose. Samples were incubated for 3.5 h at 37 °C and 4% CO_2_ atmosphere. After incubation, the cells were prepared for on-chip experiments as described above.

*Microfluidic Device:* The microfluidic chip was described in detail in Zielke et al. [42]. Briefly, the chip consists of a droplet generator where cells are encapsulated into droplets; an incubator region enabling a change in droplet pH due to cell metabolism; and a sorting region (Figure S1). Cellular solution was injected onto the chip through an aqueous inlet. Via a flow focuser, droplets were generated in 0.1% *w*/*w* Picosurf-1 surfactant oil (Sphere Fluidics Limited, Cambridge, United Kingdom) in Novec 7500. Droplet diameter was approximately 70 µm (height 25 µm) and a corresponding volume of about 80 picoliters. An additional oil outlet after droplet generation was set to a flow in the opposite direction of the main flow to reduce the amount of oil before the droplets entered the incubator region. This enabled the tight packing of droplets within the incubator to ensure the same incubation times for all droplets [45]. Average incubation time ranged from 6 to 8 min depending on the experiment before the channel narrowed and droplets entered the sorting region. At the end of the incubator, the oil solution was exchanged with QX100 oil (QX100 droplet generation oil for probes, Biorad, Hercules, CA, USA). Droplets entered the sorting region that included a rail of higher channel height (Figure S2). The rail, oriented at 45 degrees to the flow direction, allowed for the sorting of droplets by interfacial tension and hence pH. A short horizontal section of the rail served to direct droplets to the angled section of the rail, improving sorting performance as more droplets followed a similar flow path.

Flows within the chip were controlled via a computer-controlled syringe pump system (Nemesys, Cetone, Korbussen, Germany). Typical flow conditions can be found in the Supplemental Table S1. The temperature of the chip during experiments was maintained at 37 °C using a heating stage with control module and temperature feedback (CHS-1 heating plate, TC-324C temperature controller, Warner Instruments, Hamden, CT, USA).

Images and videos were taken on an inverted fluorescence microscope (Olympus IX-51, Olympus, Tokyo, Japan) equipped with a 4× objective, a shuttered LED fluorescence excitation source (Spectra-X light engine, Lumencor, Beaverton, OR, USA) and a high-speed camera (VEO-410, Vision Research, Wayne, NJ, USA). The microscope filter cube contained a dual-edge dichroic mirror (Di03-R488/561-t1-25 × 36, Semrock, IDEX Health & Science LLC, Rochester, NY, USA) and dual-band emission filter (FF01−523/610-25, Semrock, NewYork, NY, USA) that enabled transmission of both pyranine and Calcein AM fluorescence. To determine droplet pH values, the excitation source with individually addressable LEDs was coupled to an Arduino (Arduino LLC, Scarmagno, Italy) to allow for rapid alternation between different colored LEDs using simple TTL triggering. Droplets were excited with alternating violet (395 nm BP 25 nm), blue (440 nm BP 20 nm) and green excitation (561 nm BP 14 nm) at a rate of 100 frames per second (33 fps for each color).

*Data Analysis:* ImageJ software was used for image analysis [46]. The pH values of individual droplets were determined at the end of the incubator before droplets entered the sorting region via the ratio of fluorescence intensity from background-subtracted blue and violet excitation. A calibration curve from fluorescence ratios of droplets of known pH was used to determine pH using a procedure described previously [42]. Droplets containing two cells were rare and were excluded from the analysis. Green excitation was used to identify cells labeled with Calcein AM. Logistic regression was used to statistically estimate optimal pH thresholds to separate selected from non-selected cells. The pH threshold was defined at a 50% predicted probability of selecting the cell. The standard error of the prediction was used to obtain a 95% confidence interval around that threshold.

*Flow cytometry:* The cells were prepared as described above. After activation, the cells were incubated in media overnight to allow for the display of CD69 surface markers specific for activation. The next day, cells were fixed with 4% paraformaldehyde (32% solution, EM grade, Electron Microscopy Science, Hatfield, PA, USA) in PBS for 15 min at RT and washed twice with PBS before staining with Human CD69 APC conjugate (Life Technologies, Frederick, MD, USA). Staining was performed at a *v*/*v* ratio of cell suspension (~1 × 10^6^ cells/mL) to CD69 staining solution of 100:5 for 30 min on ice. After washing, the cells were resuspended in PBS and analyzed in a BD Accuri C6 Plus Flow Cytometer (BD Biosciences, San Jose, CA, USA). Cytometry files were then analyzed using FlowJo software v.10.8.1 (FlowJo, Ashland, OR, USA).

## 3. Results and Discussion

Droplet microfluidics has been shown to be a powerful tool for both single-cell analysis and sorting [36,47,48]. A major advantage of this technique is that cell secretions remain confined in the picoliter droplet and thus associated to the cell and at high concentration. These features of droplet microfluidics are leveraged in a label-free and passive sorting method, SIFT, developed in our lab [39,40]. With a specific surfactant/oil combination (QX100 droplet generation oil for probes) a change in pH leads to a concurrent increase in droplet interfacial tension. This allows for the separation of cells based on single-cell glycolysis.

**Figure 1:**
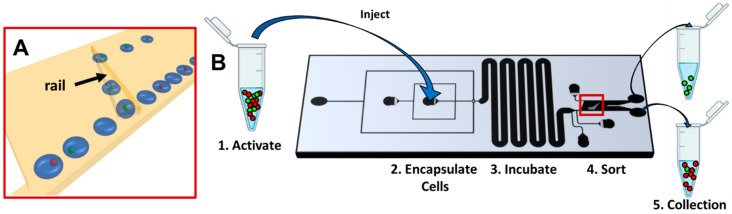
(**A**) Schematic of SIFT sorting mechanism for the population of cells with low (red) and high (green) metabolism. Empty droplets or those containing low metabolism cells are only slightly deflected by the rail. Droplets containing cells with high metabolism are separated as they follow the rail laterally. (**B**) Workflow of the sorting of activated T-cells. Naive T-cells are represented in red and activated T-cells in green. Sorting region indicated by red rectangle.

Figure 1A demonstrates the sorting mechanism for two cell populations with either low glycolysis (red) or high glycolysis (green). Cells are encapsulated and incubated. After incubation, droplets containing cells with higher glycolysis attain a lower droplet pH and concurrent increase in droplet interfacial tension. Droplets encounter a tapered rail [49], a trench with increased height oriented at 45 degrees relative to the direction of flow. The flattened, pancake-shaped droplets expand in the rail. In the case of droplets of low pH and hence high interfacial tension, they follow the rail laterally and exit near the tapered end. In contrast, in the case of droplets of higher pH and hence lower interfacial tension, the drag force of the oil immediately pushes the droplets off the rail. Hence, empty droplets or those containing low metabolism cells are only slightly deflected by the rail. The flow rate in the sorting region can be controlled by the user. This provides an independent parameter that determines the droplet pH threshold for droplet selection. This strategy can sort droplets by pH in the range of pH 6.0–7.5 [40,42]. It is used here as a passive sorting strategy to isolate highly activated T-cells.

The general scheme for the proof-of-concept isolation of activated T-cells is shown in Figure 1B. Prior to injection onto the chip, a population of T-cells is activated with beads and is mixed with a population of naive T-cells. The naive cells are fluorescently labeled so the two populations of cells can be distinguished on-chip. The cell solution is injected on chip and cells are encapsulated in droplets. Cell concentration is kept dilute to avoid multiple occupancy in the droplets. Droplets are incubated as they flow for several minutes through a wide serpentine channel to allow a change in droplet pH due to proton secretion. This change in droplet pH can be tuned by adjusting the concentration of buffer used in the droplet solution. Droplets then enter the sorting region where the rail sorts droplets by their pH. Two chip exits allow for separate collection of the unselected and selected droplets.

**Figure 2:**
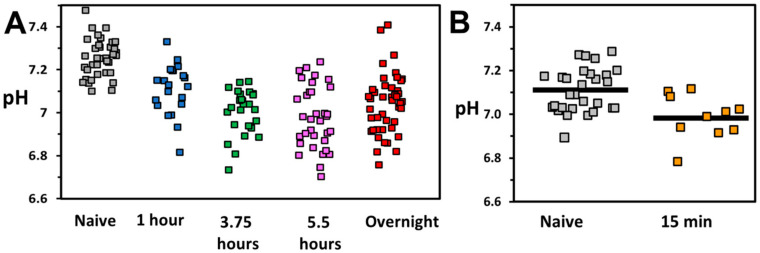
(**A**) pH of droplets containing Jurkat T-cells plotted vs. activation time with activator beads. Each marker represents the pH of one droplet with one encapsulated cell. Grey markers represent naive cells which were not exposed to activator beads. Ordinary one-way ANOVA with Dunnett’s multiple comparisons test: 1 h (*p* = 0.0002), 3.75 h (*p* < 0.0001) 5.5 h (*p* < 0.0001), overnight (*p* < 0.001). (**B**) pH of cells exposed to 15 min of activation compared to naive cells. Horizontal bar represents average pH value. Two-tailed Student’s *t*-test (*p* = 0.0025).

The pH of the droplets containing Jurkat T-cells were first characterized after incubation as a function of activation time, the time of the exposure of cells with the activation beads (Figure 2A). Cultured Jurkat cells, rather than donor CD4+ cells, were used for the time series as they provide easier preparation and manipulation. The incubation time on chip was held constant for all experiments (8 min). The pH of individual droplets was determined using a ratiometric fluorescent sensor, pyranine, as described previously [42]. Naive cells, not exposed to activation beads, had an average droplet pH of 7.29 ± 0.02 (average ± error of the mean), lower than the pH of empty droplets (7.39 ± 0.04) that remain near the initial buffer pH. The variability in pH values for naive T-cells (standard deviation of 0.10) highlights the heterogeneity of cell glycolysis for individual cells. Droplets containing activated Jurkat T-cells displayed, on average, lower pH values. After 1 h of activation, average droplet pH values were 7.12 ± 0.03. For longer activation times, droplet pH values decreased further and stabilized near an average pH of 7.00. The lower pH values for droplets containing activated cells were indicative of an increase in glycolysis upon activation. To confirm, cells with different activation times were analyzed for the surface marker CD69, a conventional indicator of early activation (Figure S3).

Average droplet pH values, however, provide a limited picture of the overall population of activated and naive cells. More relevant for single cell sorting by SIFT is that no droplets containing naive cells attained a pH below 7.10. In contrast, droplets lower than this pH represent 40% of cells activated for 1 h and from 65–85% of cells activated for longer times. Thus, by targeting this population of droplets of lower pH, we will not only largely exclude naive cells but also select cells that are highly glycolytic.

The droplet pH values for short activation time with beads (15 min) are presented in Figure 2B. Even for this short incubation time, activated cells show on average a lower droplet pH of 6.99 ± 0.03 as compared to 7.11 ± 0.02 for naive cells. This fast change is consistent with bulk measurements of extracellular acidification made on metabolic profilers [7,22]. In the droplets containing naive cells, only a single droplet displays a pH below 7.00 (3% of cells). In contrast, for activated cells this population represents about half of all droplets. A similar change in pH between droplets containing naive and activated cells was observed not only using activation beads but also through activation with soluble complexes (Figure S4). In this case, only activated cells attain a droplet pH below 7.2. The change in droplet pH upon activation was largely impeded by the treatment of cells with a glycolysis inhibitor, 2-deoxy-D-glucose (2DG), confirming that the change was indeed associated with cellular glycolysis (Figure S5). The difference in droplet pH between naive and activated cells can be leveraged to enrich highly-activated T-cells.

**Figure 3:**
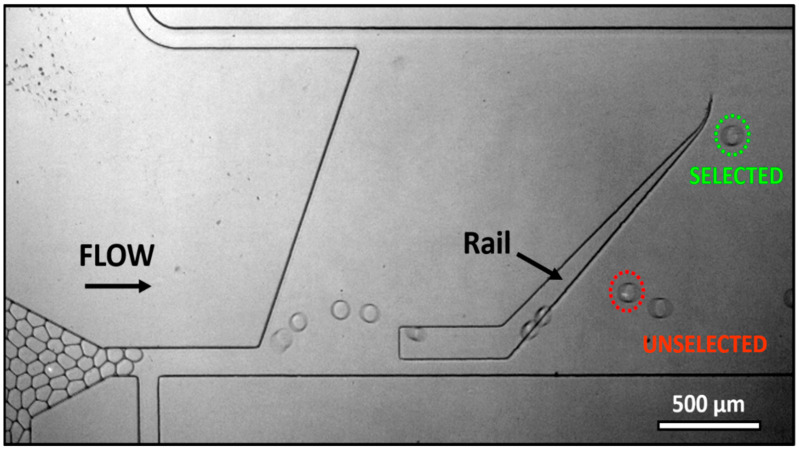
SIFT device sorting of activated and naive Jurkat T-cells. A droplet containing an activated cell (circled in green), at pH 7.10, rides the trench laterally up (selected). A droplet containing a naive cell (circled in red) with higher pH, pH 7.19, is only slightly deflected by the rail (unselected). Empty droplets are also directed to the unselected chip exit.

Figure 3 (and supplemental Video S1) demonstrates the sorting of an activated T-cell and naive cell using the SIFT technology. The droplet containing the naive cell, determined to be at pH 7.19 after incubation, is only slightly deflected by the rail. Empty droplets are also directed to the unselected exit. In contrast, a droplet containing an activated cell (15 min activation time), at pH 7.10, rides the trench laterally up and is thus separated from other droplets. This droplet is considered a selected droplet.

**Figure 4:**
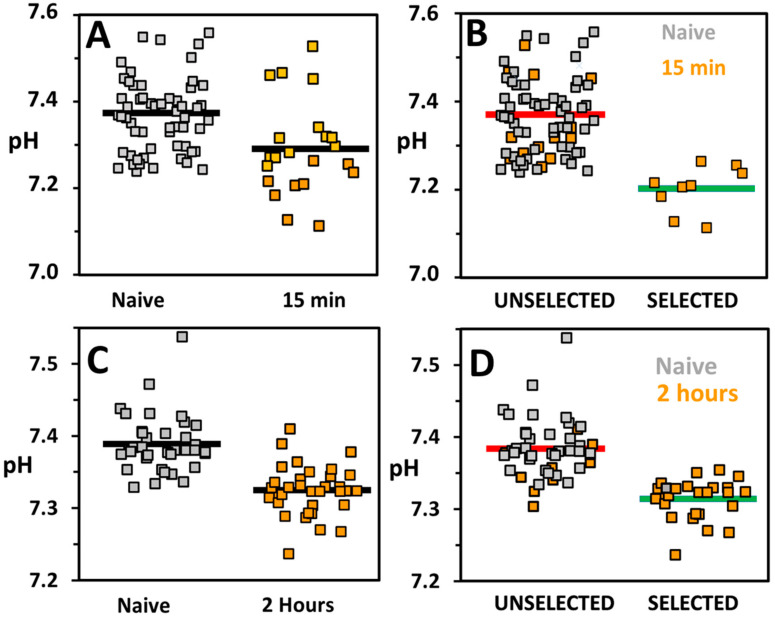
(**A**) pH of droplets containing CD4+ T-cells that are either naive (gray) or activated for 15 min (orange). Average pH values, indicated by a horizontal line, are 7.37 ± 0.01 (*n* = 59) for naive cells and 7.26 ± 0.04 (*n* = 22) for activated cells (**B**) Droplet presented as unselected and selected populations after sorting using SIFT device. While activated cells make up 27% of cells prior to sorting, selected cells contain exclusively activated cells. (**C**) pH of droplets containing naive (gray) or cells activated for 2 h (orange). Average pH values, indicated by a black line, are 7.39 ± 0.01 (*n* = 37) for naive cells and 7.32 ± 0.01 (*n* = 34) for activated cells. (**D**) Droplets presented as unselected and selected populations. The sorting of droplets leads to an enrichment of activated cells from 48% before sorting to 96% of selected cells.

Figure 4 summarizes the sorting of CD4+ fresh, cryopreserved human T-cells from a healthy donor. CD4+ T-cells were activated and mixed with a naive population before injection onto the chip for sorting. For 15 min activation, the buffering concentration of the on-chip solutions was reduced from 2.5 mM to 1 mM phosphate buffer to account for the lower glycolysis for CD4+ T-cells compared to Jurkat T-cells. The lower buffer concentration ensured a substantial change in droplet pH after incubation.

The pH values of droplets with encapsulated naive and cells activated for 15 min are presented in Figure 4A. The droplets containing activated T-cells attain lower average pH (7.26 ± 0.04) than for naive cells (7.37 ± 0.01). There is substantial overlap between the pH values of the two populations. However, unlike activated cells, the naive cells do not achieve a droplet pH below 7.24. The control of flow conditions on chip allows a selection of droplets below this pH.

Figure 4B presents the activated and naive cells as a function of selected and unselected cells. Prior to sorting, the cell population contains 27% activated T-cells. The selected droplets contain exclusively activated cells with a droplet pH range of 7.11–7.26. While the unselected population includes 22% activated cells, the activated T-cells within the selected population represent droplets with a lower pH, with an average pH of 7.20 versus 7.35 in the unselected population.

The pH threshold, the pH value at which there is an equal probability that a cell is selected or unselected, can be determined by fitting the data to a logistical regression (Figure S6A). The pH threshold was determined to be 7.24. This selection threshold can be modified by the user via a change in flow rate in the sorting region allowing for more or less stringent sorting criteria [42]. The fit also provides a measure of the resolution in pH for accurate sorting from the standard error of the prediction at 95% confidence (light blue shading in Figure S6). The pH range was determined to be 7.19 to 7.25. This error is consistent with previous measurements on the device [42].

SIFT sorting was also performed on CD4+ cells activated for 2 h. Naive cells have an average pH value of 7.39 ± 0.06 compared to 7.32 ± 0.05 for cells activated for 2 h (Figure 4C). The lowest droplet pH value for a naive cell is 7.33. In contrast, more than half (52%) of all activated cells attained pH values below 7.33. On average, pH values are higher than those observed for 15 min activation. This can be explained by the use of higher phosphate buffer in these experiments (2.5 mM vs. 1mM).

Figure 4D shows the naive and activated cells as a function of selected and unselected populations. The enrichment of activated T-cells from 48% to 96% was obtained from the initial to selected population (Figure 4D). In contrast, the unselected cell population contained 20% activated cells. The pH threshold was determined to be 7.34 ± 0.01 (threshold ±95% confidence interval) (Figure S6B).

In the above experiments, the mixed population of naive and activated cells was enriched to over 95% activated cells. Moreover, as extracellular acidification rate can be correlated to level of activation, [7] the technique can be used to select the subpopulation of cells with the strongest activation. As activation strength is also correlated to cell fate [5], the selected highly glycolytic population may also favor differentiation to pro-inflammatory Th1 cells. For these experiments, the flow conditions were chosen to set a threshold just below the pH of droplets in the population of naive cells and led to the selection of approximately 40% of the activated cells. The threshold could be set to either further exclude naive cells or to capture a small percentage of highly activated T-cells for study or use. The phosphate buffer used in experiments was either 1 mM or 2.5 mM. The lower buffer concentration ensured a spread of pH values even for short activation times (Figure 4A). This parameter can be tuned based on the glycolysis levels of the cells under study. Although the focus here was on the sorting of cells shorty after activation, metabolic reprogramming after activation is long-lasting (Figure 2A), persisting even for several days. This would mean that highly-glycolytic activated cells could be isolated long after the initial activation event.

The data presented in Figure 4 represent 2 min of collection time with the SIFT device and a corresponding video file consisting of approximately 12,000 frames. Droplets were sorted at a rate of about 12–20 Hz. Cell density was kept low (1 in 20–30 droplets contain a cell) to avoid multiple occupancy of cells in droplets. This collection time was ultimately limited by video collection that was necessary here for the determination of pH and validation of sorting accuracy. Without this constraint, longer collection times would allow the sorting of hundreds of cells. To recover cells after sorting, droplet breakup can be initiated by de-emulsifier chemicals, [50] dilution of surfactant [51] or electrostatic charge [52]. The surfactant in oil used in the sorting region (Droplet generation oil for probes) may have detrimental effects on cells [39,53]. However, exposure is kept at a minimum by introducing this chemical only after incubation. Furthermore, the addition of Pluronic F-68 to the droplet media also promotes cell viability. Breaking-up of droplets immediately upon collection off-chip leads to viable cells as determined by trypan blue and Calcein AM assays.

## 4. Conclusions

The SIFT technique presents a method to isolate activated cells with the highest glycolysis from both naive cells and activated cells with a lower metabolism. The activation and differentiation of T-cells leads to a complex metabolic reprograming that serves both to initiate and regulate these events. These events are further impacted by cell environment and nutrient availability [24,25]. The full understanding of the coordination of these events and differentiation outcomes has been limited by tools that can select single cells by their metabolic profiles. Thus, by selecting cells by single-cell glycolysis, prior to the display of surface markers, SIFT presents a method to help detangle this complexity especially when combined with downstream analysis tools. Moreover, since the pH change may be correlated to the level of activation, this technique allows for the isolation of T-cells with the earliest activation and metabolic reprogramming for the testing of T-cell activation modulators. Indeed, the isolation of T-cells based on their metabolism rather than conventional activation markers is relevant to multiple aspects of immunometabolism and downstream applications in T-cell biology. Immunotherapy and co-receptor manipulations have impacts on activation-induced glycolytic metabolism, [54] and regulators of T-cell differentiation may also act via the fine-tuning of early metabolic signaling.

The presented SIFT technology is both a label-free and passive sorting method. It offers potential benefits in simplicity and cost. By transposing cell activation to a physical property, droplet interfacial tension, the technique forgoes the components associated with the fluorescent sorting of T-cells such as labels, light sources, detectors, and active sorters. This simplicity facilitates correlating glycolytic activity with cell markers using the fluorescence channel during sorting or more elaborate analysis (genomic, transcriptomic, proteomic) on collected cell populations. Future efforts will focus on the improvement of the device while developing a robust workflow for the study and characterization of sorted T-cells. The current chip design (Figures S1 and S2) has a maximum throughput of about 30 droplets per second, lower than the typical throughput of hundreds or thousands of droplets per second by active droplet sorters [36,38]. Increasing this throughput, through design or parallelization, would enable its application for isolating rare cells within a population such as antigen specific T-cells, or the most activated T-cells isolated from the tumor microenvironment.

## Supplementary Materials

The following supporting information can be downloaded at: https://www.mdpi.com/article/10.3390/mi13091442/s1, Figure S1: SIFT device channel geometry; Figure S2: Sorting rail dimensions and position.; Figure S3: Flow cytometry analysis for CD69 expression; Figure S4: Droplet pH for ImmunoCult Activation; Figure S5: Droplet pH for 2-deoxy-D-glucose (2DG) treatment; Figure S6: Logistic Regression Fits; Table S1: Typical flow parameters; Video S1: SIFT cell sorting.
