# Supplementary material for: Droplet Microfluidic Technology for the Early and Label-Free Isolation of Highly-Glycolytic, Activated T-Cells
Source: Micromachines (Basel). 2022 Sep 1;13(9):1442. doi: 10.3390/mi13091442 (PMC9503730; doi:10.3390/mi13091442)

Droplet Microfluidic Technology for the Early and Label-free Isolation of Highly-Glycolytic, Activated T-cells

Claudia Zielke,[1] Adriana J. Gutierrez Ramirez,[1] Kelsey Voss[2], Maya S. Ryan,[1] Azam Gholizadeh,[1] Jeffrey C. Rathmell[2,3], Paul Abbyad[1*]

1 Department of Chemistry and Biochemistry, Santa Clara University, Santa Clara, CA, 95053, USA

2 Department of Pathology, Microbiology, and Immunology, Vanderbilt University Medical Center, Nashville, TN, USA

3 Vanderbilt Center for Immunobiology, Vanderbilt University Medical Center, Nashville, TN, USA

* corresponding author: pabbyad@scu.edu

Supplemental Information

Table of Contents:

Video S-1…...……………………………………………………………..…...Page S-2

Figure S-1…...……………………………………………………………..…..Page S-3

Figure S-2…...……………………………………………………………..…..Page S-4

Figure S-3…...……………………………………………………………..…..Page S-5

Figure S-4…...……………………………………………………………..…..Page S-6

Figure S-5…...……………………………………………………………..…..Page S-7

Figure S-6…...……………………………………………………………..…..Page S-8

Table S-1…...……………………………………………………………..…....Page S-9

Video Caption:

Video S1. SIFT cell sorting. The video demonstrates sorting using SIFT of droplets containing activated and naive Jurkat cells. Video shows the end of incubator and sorting region.

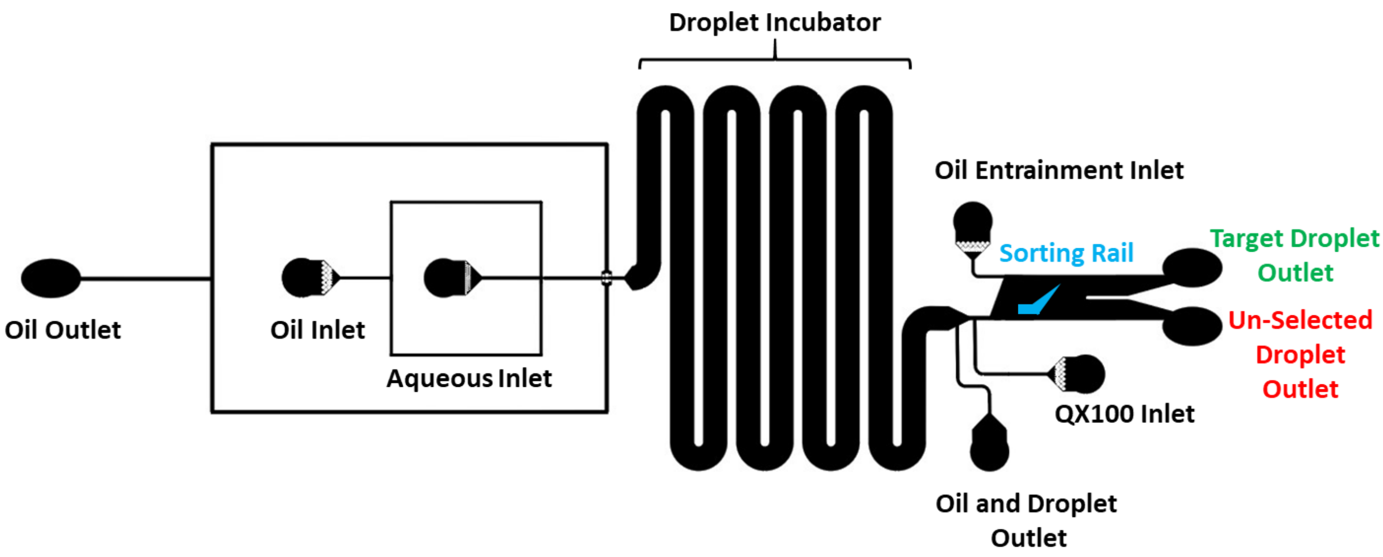

Supplemental Figure S1. SIFT device channel geometry.

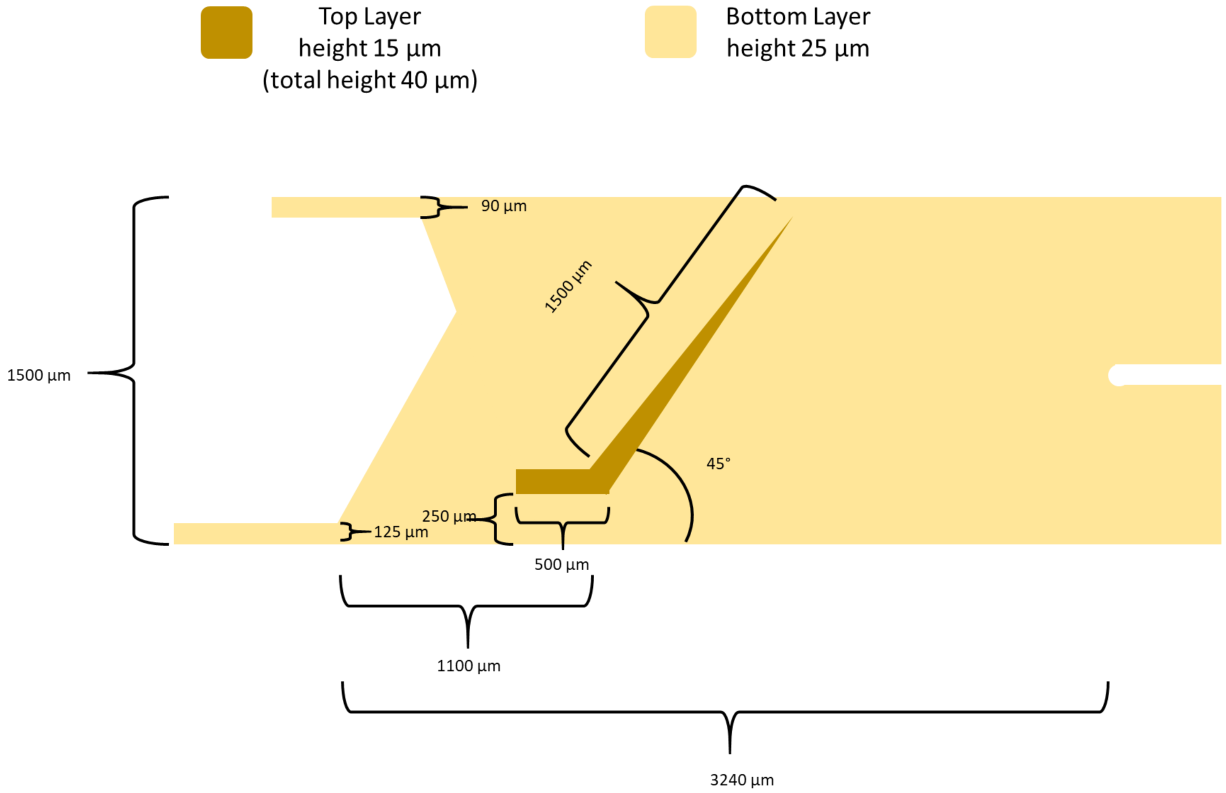

Supplemental Figure S2. Sorting rail dimensions and position. Exact position of rail is approximate as layers are positioned by eye.

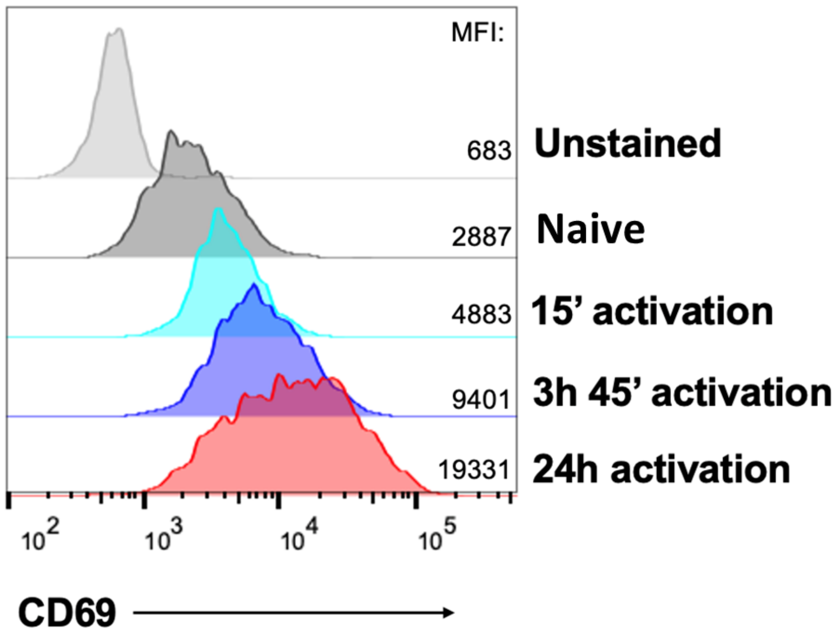

Supplemental Figure S3. Flow cytometry analysis for CD69 expression. Jurkat T-cells were activated for variable times with anti-CD3/CD28 activation beads before staining with Human CD69 APC conjugate. CD69-APC expression is shown on a log scale. MFI= mean fluorescence intensity.

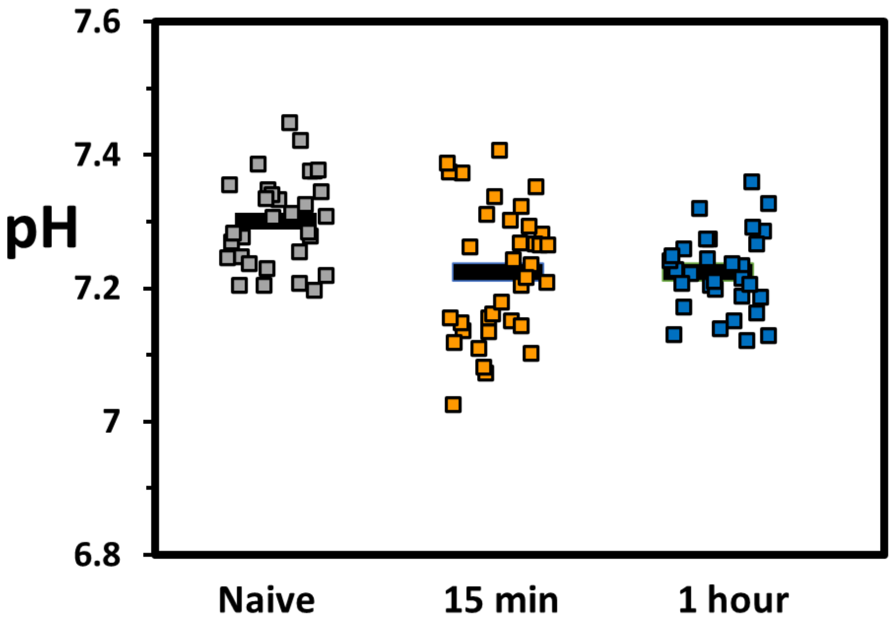

Supplemental Figure S4. Droplet pH for ImmunoCult Activation. pH of droplets containing Jurkat T-cells plotted vs activation time with ImmunoCult soluble activation complexes. Each marker represents the pH of one droplet with one encapsulated cell. Grey markers represent naive cells which were not exposed to soluble activation complexes. Average pH values, indicated by a horizontal line, are 7.30 ± 0.01 (*n*= 31) for naive, 7.22 ± 0.02 (*n*= 36) for 15 min activation and 7.22 ± 0.01 (*N*= 31) for 1 hour activation.

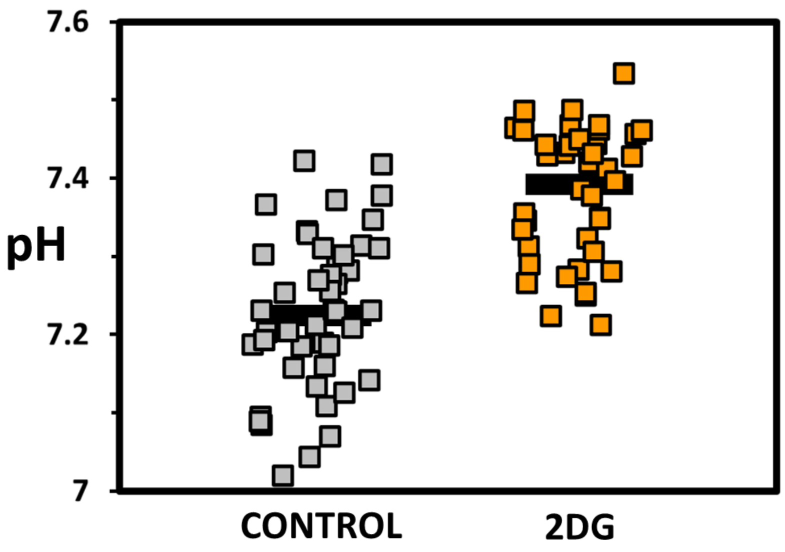

Supplemental Figure S5. Droplet pH for 2-deoxy-D-glucose (2DG) treatment. pH of droplets containing Jurkat T-cells treated for 3.5 hours with 100mM of 2-deoxy-D-glucose (2DG) prior to activation for 15 minutes with anti-CD3/CD28 activation beads (orange). Grey markers represent control cells which were kept in the same conditions but with low glucose media supplemented with 100mM of glucose rather than 2DG prior to activation. Both control and 2DG treated cells were activated in low glucose media. Average pH values, indicated by a horizontal line, are 7.39 ± 0.01 (*n*= 60) for control cells and 7.22 ± 0.01 (*n*= 60) for 2DG treated cells.

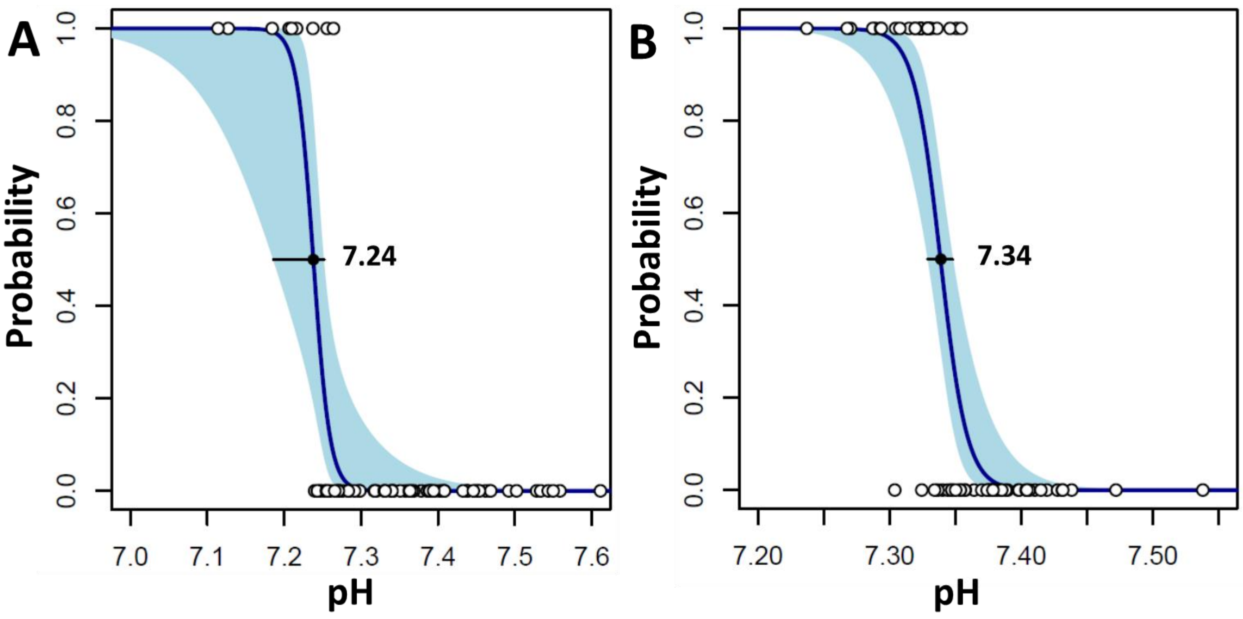

Supplemental Figure S6. Logistic Regression Fits (**A**) Logistic regression fit of binary selected/unselected data with pH for CD4+ cells activated for 15 minutes with beads (**B**) Logistic regression fit for CD4+ activated for 2 hours. pH thresholds are indicated on graph and represent where there is equal probability that droplets are selected or unselected. The 95% confidence limit is indicated in light blue.

Supplemental Table S1. Typical flow parameters. Channel geometry is provided below for reference. Negative flows below are opposite in direction to the main flow in the channel.

Inlets and Outlets	Flow Rates (µL/min)
Aqueous Inlet	0.1 - 0.5, most commonly 0.3
Oil Inlet	3 – 5, most commonly 3
QX100 Inlet	2 – 5
Oil Entrainment Inlet	8 – 20
Oil Outlet	- 2.7 to -4.7
Oil and Droplet Outlet	-0.25 to -1

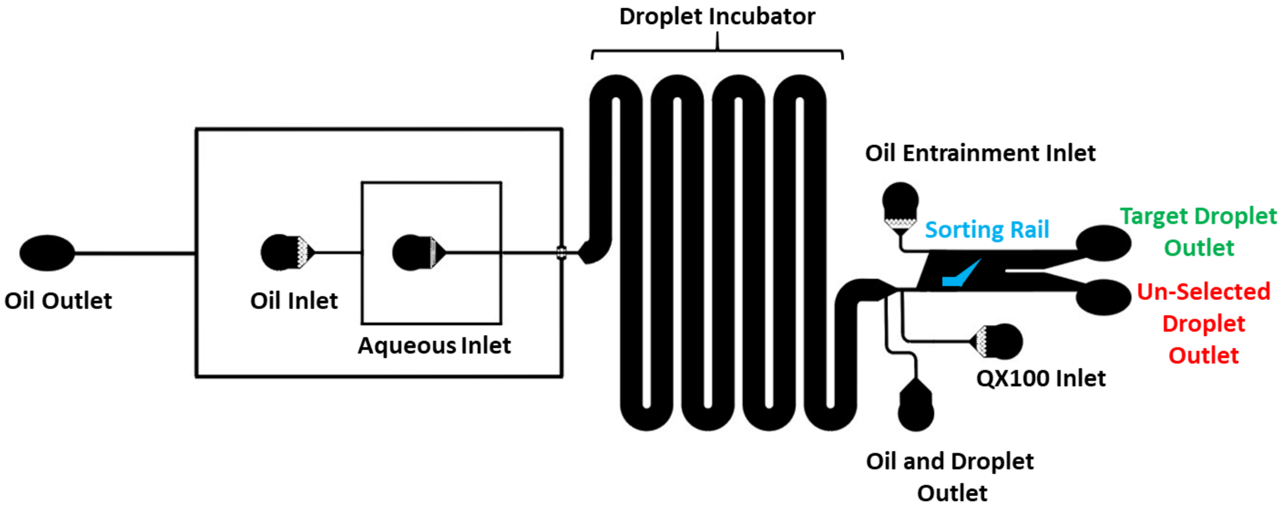

Supplement: Supplementary file 1 [file micromachines-13-01442-s001.zip › T-Cell Sorting SI PROOFS.pdf]
